# Editorial: The development of health professional educators

**DOI:** 10.15694/mep.2018.0000069.1

**Published:** 2018-04-02

**Authors:** Gary D. Rogers

**Affiliations:** 1 School of Medicine and Health Institute for the Development of Education and Scholarship (Health IDEAS)

**Keywords:** Faculty development, MedEdPublish special issue, Health professional educators

## Abstract

This article was migrated. The article was marked as recommended.

No abstract.

## Introduction

*No bubble is so iridescent or floats longer than that blown by the successful teacher*

( Osler, 1911)

Educators in the health professions are facing an increasingly challenging environment. They are tasked with supporting the development of effective practitioners in the face of shrinking resources, over-stretched care systems and a political environment that privileges countable health services for citizens of the present over clinical learning that will benefit the citizens of the future. Ensuring that educators have, and can continue to develop, the capabilities required to plan, facilitate and assess the learning of health professional students, trainees and practitioners is an important challenge in a changing world.

This special issue of *MedEdPublish* will concentrate on the processes of developing those charged with the education of health professionals, across the spectrum from university-based teachers and educationalists to clinicians who facilitate learning in health practice settings and providers of continuing professional development for qualified practitioners. It is focused at the ‘meta-’ level of enhancing the learning of those who help others to learn.

## The development of health professional educators

This area of work is almost synonymous with ‘faculty development’, which Steinert (2014, p. 4) has defined as ‘all activities health professionals pursue to improve their knowledge, skills, and behaviours as teachers and educators, leaders and managers, and researchers and scholars, in both individual and group settings’. Her definition does not quite encompass all that the issue seeks to cover, however, since not all of the individuals who contribute significantly to the education of health professionals are themselves health professionals. Other experts also require expert assistance to develop and refine their capabilities to optimise the learning of health care workers-in-training. These include education scholars who work in health, specialists in related professions such as health scientists and health care lawyers and, very importantly, the specifically-trained performers, known as ‘standardized patients’ in North America and ‘simulated patients’ in most of the rest of the world, who contribute significantly to the education of health professionals. Further, this edition will also focus particularly, within the broad scope of ‘faculty development’, on the relatively neglected development of *educational* capability, rather than the skills and knowledges related to research and leadership included in Steinert’s definition.

Traditionally, there had been a naïve expectation that content experts would also automatically be good educators but from around the middle of the 1970s in general higher education, institutions in the United States began to develop and implement formal programs to improve the teaching abilities of their academic staff ( Lewis, 1996). In medicine, attention to developing the educational capabilities of teachers has a more complex history with evidence of scholarly activity related to this area as early as the beginning of the 1950s (e.g. Shapiro, 1951) and a cross over with the formulation of ‘medical education units’ across the developed world in the 1970s (Davis, Karunathilake & Harden, 2005; Al-Wardy, 2008). Only more recently has this effort expanded to include educators the other health professions ( Scheckel, 2009; Boyce et al, 2008).

Since the turn of the current century, there has been a rapid expansion of work in the area, as well as a shift from a focus only on formal educational activities towards a more comprehensive view of educator development. This wider perspective recognises the agency of teachers in their own education and the importance of critical reflection ( Sullivan & Irby, 2011). It also encompasses the promotion of self-directed teacher learning and scholarship, as well as recognition programs (such as Fellowship programs offered by AMEE, the international medical education organisation, and the Australian and New Zealand Association for Health Professional Educators, as well as the work of the Academy of Medical Educators in the UK and local ‘academy’ programs at several US institutions). During this period, four biennial International Conferences on Faculty Development in the Health Professions (the most recent in Helsinki in 2017) have provided an opportunity for scholars and practitioners in this area to share ideas and forge cross-country collaborations. Presenters at these conferences who have not yet published their work are particularly invited to submit contributions to this special edition of *MedEdPublish.*

In 2017, AMEE constituted a formal Faculty Development Committee with terms of reference that include ‘To advance best practices and excellence in the development of health professional academic faculty across the globe’. Accordingly, the special edition provides a timely opportunity to further this aim by sharing your scholarly work and practice experience in this important area of endeavour.

## Take Home Messages

*MedEdPublish* provides a unique opportunity for both experienced and emerging scholars in health professional education to publish their work for the benefit of our broad practice community. In this special edition, I invite you to share your research in, and experience of, supporting and developing health professional educators. I would welcome contributions from everyone interested in publishing their work on the training, mentorship and continuing professional development of those who facilitate the learning of health professionals, including academic faculty, small group tutors, clinical supervisors and simulated patients. Contributions are welcome from authors with a primary focus on faculty development, as well as from teachers ‘in the field’ on what additional education and support they need to work to their full potential.

## Notes On Contributors

Gary D. Rogers MBBS, MGPPsych, PhD, FAMEE, FANZAHPE, PFHEA is Professor of Medical Education Deputy Head (Learning & Teaching) of the School of Medicine, Griffith University, Queensland, Australia, as well as a role as Program Lead for Interprofessional and Simulation-Based Learning in the Griffith Health Institute for the Development of Education and Scholarship (Health IDEAS).

## Declarations

Professor Gary D. Rogers is the guest Theme Editor for the AMEE MedEdPublish theme - The development of health professional educators.
