# Supplementary material for: Editorial: The development of health professional educators
Source: MedEdPublish (2016). 2018 Apr 2;7:69. [Version 1] doi: 10.15694/mep.2018.0000069.1 (PMC10711941; doi:10.15694/mep.2018.0000069.1)
Supplement: Peer review history

# Reviewer report: Reviewer response for version 1

**DOI:** 10.21956/mep.19542.r28948

**Published:** 2018-05-01

**Authors:** Varsha Patel

**Affiliations:** 1 NHL Municipal Medical College

This review has been migrated. The reviewer awarded 5 stars out of 5 Being involved in faculty development, I highly appreciate the theme. Professor Rogers has set the ball rolling for all who wish to share something worthwhile on this platform and that too in a concise manner.

Reviewer Expertise:

NA

No decision status is available

# Reviewer report: Reviewer response for version 1

**DOI:** 10.21956/mep.19542.r28947

**Published:** 2018-04-08

**Authors:** Subha Ramani

**Affiliations:** 1 Harvard Medical School, Brigham and Women's Hospital

This review has been migrated. The reviewer awarded 5 stars out of 5 Professor Rogers is absolutely correct that developing health professions educators and educational leaders is a challenging endeavor. I agree that content experts cannot carry out this obligation effectively and the system needs and has utilized education experts to do so. One might question this strategy to inquire whether educational expertise alone can fully comprehend the context within which health professions educators practice. The health care environment is unique, hectic, often chaotic, beset by many obstacles- expected and unexpected, new regulations, shrinking finances etc. Thus, it is wonderful to see more and more health professionals seek training in education, collaborate with non health professional educators and design education to improve health care practices. I look forward to this special issue to see what educational strategies and programs have been developed around the world to improve health professions education and ultimately patient care.

Reviewer Expertise:

NA

No decision status is available

# Reviewer report: Reviewer response for version 1

**DOI:** 10.21956/mep.19542.r28950

**Published:** 2018-04-04

**Authors:** Nandalal Gunaratne

**Affiliations:** 1 Faculty of Medicine, Wayamba University of Sri Lanka

This review has been migrated. The reviewer awarded 5 stars out of 5 It is good to see interest in the education specialist being recognised. Hoping to continue to learn and improve with your initiative.

Reviewer Expertise:

NA

No decision status is available

# Reviewer report: Reviewer response for version 1

**DOI:** 10.21956/mep.19542.r28949

**Published:** 2018-04-02

**Authors:** Trevor Gibbs

**Affiliations:** 1 AMEE

This review has been migrated. The reviewer awarded 5 stars out of 5 An interesting theme about a very important subject. When the first Tomorrows Doctors document was published in 1993, I am sure that there were many you felt assured that interprofessional learning was on the agenda, but much fewer who wondered how we were going to do it- indeed we are still widely exploring this issue to this very day. I would imagine there were only a handful of people, in the bigger debate, who had considered the education of those faculty designed to educate the health professionals of the future, or if they had their own ideas and innovations to share. Hence I think this call for papers has come at a very opportune moment. I am looking forward to learning from these papers as I am sure we all will

Reviewer Expertise:

NA

No decision status is available

# Reviewer report: Reviewer response for version 1

**DOI:** 10.21956/mep.19542.r28946

**Published:** 2018-04-02

**Authors:** Mohammed Osman

**Affiliations:** 1 General orgainization for teaching hospitals

This review has been migrated. The reviewer awarded 5 stars out of 5 A very interesting article. The field of medical education is growing so fast and its scope is expanding significantly. This requires changes in policies, curriculum, training, mentorship,........ Here is the role of research. I beleive there is a great role on well reputated publications, such as, MedEdPublish ‘To advance best practices and excellence in the development of health professional academic faculty across the globe’. As well as that to motivate authors from all over the globe to submit their work in this glowing field.

Reviewer Expertise:

NA

No decision status is available
